# Overcoming Challenges of Applying Reinforcement Learning for Intelligent Vehicle Control

**DOI:** 10.3390/s21237829

**Published:** 2021-11-25

**Authors:** Rafael Pina, Haileleol Tibebu, Joosep Hook, Varuna De Silva, Ahmet Kondoz

**Affiliations:** Institute of Digital Technologies, Loughborough University London, 3 Lesney Avenue, London E20 3BS, UK; h.tibebu@lboro.ac.uk (H.T.); j.hook@lboro.ac.uk (J.H.); v.d.de-silva@lboro.ac.uk (V.D.S.); a.kondoz@lboro.ac.uk (A.K.)

**Keywords:** vehicle control, reinforcement learning, curriculum learning, sim-to-real world, intelligent mobility

## Abstract

Reinforcement learning (RL) is a booming area in artificial intelligence. The applications of RL are endless nowadays, ranging from fields such as medicine or finance to manufacturing or the gaming industry. Although multiple works argue that RL can be key to a great part of intelligent vehicle control related problems, there are many practical problems that need to be addressed, such as safety related problems that can result from non-optimal training in RL. For instance, for an RL agent to be effective it should first cover all the situations during training that it may face later. This is often difficult when applied to the real-world. In this work we investigate the impact of RL applied to the context of intelligent vehicle control. We analyse the implications of RL in path planning tasks and we discuss two possible approaches to overcome the gap between the theorical developments of RL and its practical applications. Specifically, firstly this paper discusses the role of Curriculum Learning (CL) to structure the learning process of intelligent vehicle control in a gradual way. The results show how CL can play an important role in training agents in such context. Secondly, we discuss a method of transferring RL policies from simulation to reality in order to make the agent experience situations in simulation, so it knows how to react to them in reality. For that, we use Arduino Yún controlled robots as our platforms. The results enhance the effectiveness of the presented approach and show how RL policies can be transferred from simulation to reality even when the platforms are resource limited.

## 1. Introduction

Reinforcement learning has been well studied in the recent past as it is considered one of the most prominent paradigms in machine learning [1,2]. Inspired by the biological behaviours of humans or animals, RL consists in following a trial-and-error basis to train the agents to learn the best actions for each situation they face [3]. RL quickly proved to be useful in the gaming industry, where famous works have proved how a RL agent was capable of beating the human being in games such as Go or in a set of the famous Atari games [4,5].

Due to its proven success, the areas of application of RL have expanded to diverse fields. For instance, in healthcare studies have found RL to be useful as an assistant for critical decision making [6] or in aiding in the treatment of certain conditions [7,8]. RL has also been used in finance as a tool for portfolio optimization [9] or for analysing different types of stock markets [10]. In the field of robotics control and autonomous systems the applications are also many. As one of the first applications of RL in real robots, in [11] the authors present a robot that learns how to push boxes, trained using RL. More recently, works such as [12,13] propose new methods that use RL in mobile robots and autonomous vehicles to improve on-site learning. Outside the ground domain, [14] show how RL can be applied also to the air domain by teaching flight control policies to an autonomous helicopter.

Coming back to the ground domain, when it comes to intelligent vehicle control there are multiple aspects that should be considered. Path planning is a key aspect in autonomous navigation where RL can play an important role [15]. Path planning can be described as a process that enables a mobile robot to traverse between the specific start and goal location in an environment with static and dynamic obstacles. The robot is required to find the optimal path with the shortest time, distance, and working cost. 

There are three types of path planning algorithms: traditional algorithms, intelligent bionic algorithms, and reinforcement-based path planning algorithms [16]. Traditional path planning algorithms include A* algorithms and Dijkstra algorithms. Genetic algorithms and ant colony algorithms are typical examples of bionic path algorithms. Traditional and bionic algorithms can achieve a good result in a known environment on a global mapping scale. However, real-world road conditions are very complex. In recent years, RL has become more suitable for path planning activity because it requires less prior information about the environment [17,18]. 

In path planning there are two conventional approaches: offline and online planning. The former approach assumes that the environment is perfectly known, and obstacles are static. The latter approach assumes environments are partially known, and blocks could be dynamic [19]. Traditional offline approaches cannot be directly applied to solve online path planning problems because they assume static obstacles. 

Learning-based approaches, such as deep learning and RL, have been studied to tackle online path planning in a dynamic environment [20,21,22]. With all its shortcomings, RL is best suited for problems such as path planning because it requires low prior knowledge of the environment. In [23], the authors present for the first time how to employ RL for path planning. The focus of this work was to test the usability of RL for collision avoidance in a multi-robot environment. As a combination of deep learning and RL, deep RL has also become a popular research interest for path planning problems [15,24,25,26,27,28]. Deep Q-networks (DQNs) [5] is a deep RL algorithm which combines Q-learning [29] and deep neural networks. In the DQNs approach, it is possible to use the current state and action as an input of the neural network to get the action’s Q-value as an output. Once acquiring all possible actions, the one with the maximum Q-value will be chosen. Finally, the corresponding action is selected and executed. In [30] the authors proposed a deep RL method that decreases the learning time by parallelising the algorithm across multiple robots. However, despite Deep Q-networks notable success, DQNs have two major drawbacks: they require high computational power and a long learning time. 

While progress has been made about how to use RL in path planning tasks there are still challenges to address. For instance, when the environment is completely unknown and complex, applying RL in path planning can be challenging due to the many possible states that the agent needs to explore in order to become completely aware of the environment around [31,32]. Therefore, in this work we carry out a set of experiments to analyse the impact of the environmental complexity on the learning process, and more specifically on the learning time for path planning tasks. Additionally, we discuss a method for transferring RL policies from simulation to reality. With such setup it is possible to cover multiple situations in simulation that would be unexpected in the real-world and would make the agent confused. By doing it in simulation first, then the agent is able to react to these situations in reality accordingly.

Analysing from a multi-agent perspective, to apply these concepts in situations with multiple agents can be increasingly difficult when the number of agents is increased. Such configuration will have an impact in the complexity and stability of the environment [2]. To overcome the gap between theoretical RL and its applications to real scenarios, in this work we also explore how CL can be applied to multi-agent settings in the context of intelligent vehicle control. More specifically, we use this method in a driving decision making problem that consists of a traffic junction. The contributions of this paper are as follows:

1. We investigate the impact of the environmental complexity in the learning process of RL tasks involving path planning scenarios;
2. We discuss a method for transferring RL policies from the simulation domain to the real-world domain, supported by empirical evidence and a working algorithm for the discussed method;
3. We show how CL can be applied within the context of intelligent vehicle control in tasks involving multiple agents.

The rest of this paper is organized as follows: in section two we introduce the key concepts to the understanding of this paper and we present a review of those concepts. In section three, we describe the methodology and experimental setup used in the experiments that are illustrated and discussed in section four. Finally, we conclude this paper in section five, where we also discuss the future work following the results in this paper.

## 2. Background

### 2.1. Q-Learning

Q-learning is one of the most popular RL approaches. Therefore, it has been widely used to solve path planning problems [31,32]. Q-learning attempts to learn the theory of delayed rewards to get better future rewards that are indirectly a consequence of the previous actions. The main objective of this method is to avoid local optimal activities that are not globally aligned. Reference [33] introduced Q-learning as a Temporal Difference approach (TD). In [29], the authors proved the convergence of Q-learning to optima and its relationship with TD and dynamic programming. To overcome one of the limitations of using Q-learning for path planning tasks—the impracticality of storing the Q-table for all states—[34] proposes a real-time Q-learning approach that avoids storing tables in advance. The simulation was conducted in a 10 by 10 grid map with static obstacles. Other algorithms such as Deep Q-Networks (DQNs) [5] have also been proposed to overcome similar limitations. 

Reference [31] introduced a Q-learning approach which involves the addition of a distance aspect into the decision of direction. This approach reduced the number of steps taken by the agent. However, the time taken by the model to converge is greater than the traditional Q-learning. Reference [35] proposed a method to reduce the convergence time of Q-learning for a path planning application by introducing the Flower Pollination Algorithm (FPA) to improve the initialisation of Q-learning. This method was only tested in a simple and static environment. Reference [32] investigated the use of a single source transfer and improved Q-learning transfer to acquire better learning. While this method was tested and it reduced learning speed when compared with the conventional methods, it was only tested in a complex static environment. 

To demonstrate the use and challenges of RL in path planning applications, in this work we use the Q-learning algorithm in different environmental setups. Q-learning is a model-free representative learning algorithm. An action related to a specific state is defined by a policy. Q-learning uses this policy to select an action for the agent. The policy establishes the reward or penalty for a particular action concerning the state. Q-learning agents learn by updating their Q-function whose values are obtained from the following equation,

(1) $$Q\left(s,a\right)=r\left(s,a\right)+\gamma max_{a}\left(Q\left(s^{\prime },a\right)\right)$$

where s is the current state, a is the action, r is the reward, $s^{\prime }$ is the next state resulting from taking an action a at the state s, and γ is the discount factor. The role of the discount factor in this equation is to help defining the agent’s preference towards a short-term reward. The value of γ is between [0, 1]. While values closer to 0 make the agent susceptible to a short-term reward, a value closer to 1 makes the agent prone to a long-term reward. 

### 2.2. Multi-Agent Reinforcement Learning and Curriculum Learning

Deep Neural Networks (DNNs) are usually trained using variations of stochastic gradient descent. A subset of training data is randomly sampled from the training set, passed through the DNN, and the loss and the gradients are calculated and propagated through the layers of the DNN. Stochastic gradient descent not only increases computational efficiency for very large datasets, but it is also an unbiased estimator of the true gradient of the DNN. However, we never see human teachers open the textbook, pick random exercises from random topics, and ask their students to solve the exercises in the hopes that after enough time has passed, all the material in the textbook will be covered and the students learned the material. Given enough time, all topics may eventually get covered, but it is more likely that the students quit soon out of sheer frustration. Usually, the teacher follows a curriculum, designed to facilitate the learning of a collection of interrelated topics. For example, it would make sense to teach children addition before multiplication, because you can teach multiplication through addition. This way new knowledge is built on something they already know, minimising the amount of new material needed to learn. CL [36] is a method of structuring the learning process to facilitate learning for DNNs. Informally, CL proposes to first learn and solve simpler versions of the task at hand, gradually building up to more difficult tasks. The curriculum can be thought of as a sequence of tasks, each consecutive task at least as challenging as the previous one. Assuming there is transferable knowledge between any two consecutive tasks, we may be able to re-use existing knowledge to facilitate learning the next task. By training the DNN on intermediate tasks, we assume the learner will be better prepared to learn the target task, compared to a randomly initialised learner. The existing knowledge could help the DNN reach an acceptable level of performance faster than without using a curriculum or be able to learn a complex composite computer vision which cannot be learned well without a curriculum [37].

**Figure 1:**
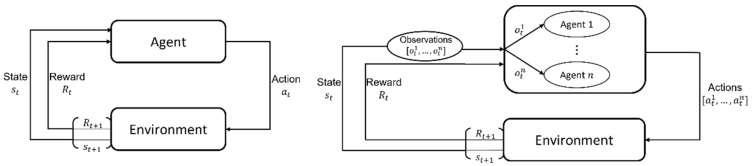
Illustration of the main differences between Reinforcement Learning (RL) (**left**) and Multi-Agent Reinforcement Learning (MARL) (**right**) (adapted from [38]). The figure represents the dynamics of a RL and MARL systems at a given time step *t*.

To create curricula, a way of generating intermediate tasks is required. In Multi-Agent Reinforcement Learning (MARL), multiple agents interact with each other in order to achieve a certain objective. When compared to single-agent RL, MARL differs mainly in the fact that there is more than one single agent involved in the task of the environment. Hence, there is a team reward that corresponds to the performance of all the agents, and they all receive this same team reward. Furthermore, there is also a set of actions corresponding to each agent instead of a single action and each individual agent may receive different state information depending on their positions in the environment. In other words, in MARL different agents may see different parts of the environment at each state. Figure 1 illustrates the main differences between single agent and multi-agent RL. Naturally, in MARL, a simple way of creating new tasks is by changing the number of agents in the system. For example, instead of training robot soccer players in a 11 vs. 11 player environment, they can first be trained in a 5 vs. 5 environment. Changing the number of agents such as this can lower the difficulty of the task (reduce the number of opponents), change the meaning of the task (a 1 vs. 5 soccer game focuses on training the goalkeeper) and lower the resource costs used to operate the (virtual or physical) learning environment. Agent Count Based Curricula have been used with success in MARL systems.

Reference [36] defines CL(CL) as a sequence of learning distributions $Q_{\left(\lambda \right)},0\leq \lambda \;\leq 1$, where:

(2) $$\forall \epsilon >0\;:\;\lambda <\lambda +\epsilon \to H\left(Q_{\lambda }\right)<H\left(Q_{\lambda +\epsilon }\right),\;\forall \epsilon >0\;:\;\lambda <\lambda +\epsilon \to W_{\lambda }\left(z\right)<W_{\lambda +\epsilon }\left(z\right)$$

where $H\left(Q_{\lambda }\right)$ is entropy H of the distribution Q at step λ, $W_{\lambda }\left(z\right)$ is the weight of training sample z in $Q_{\lambda }$. Intuitively, in each successive distribution, the training samples get re-weighted, with entropy of the distribution $H\left(Q_{\lambda }\right)$ growing monotonically. The initial distribution, $Q_{0}$, would favour “easy” samples and place a weight of 0 on “hard” samples. In the distribution sequence, the weights of all samples are gradually increased, culminating in a distribution where all samples have a weight of 1, which is the target training distribution, $Q_{1}$. However, in [36] the authors do not specify a measure for the “hardness” of samples, indicating the need for future work in this regard. In addition to formalising CL, the authors found that a learner trained with CL achieved better generalisation compared to the learner trained without a curriculum in their two experiments. Additionally, they hypothesised that CL has a regularising effect, similar to unsupervised pre-training [39], on the final learning task. In [39] the authors theorised that each successive distribution in the curriculum would guide the learner’s internal parameters (such as the weights in a neural network) toward regions in parameter space that would serve as a better starting point for the next task in the curriculum.

When it comes to multi-agent systems, recent works have focused on formulating CL specifically in the context of RL. Reference [40] defines a curriculum as a directed acyclic graph, where the edges of the graph introduce a partial ordering over the nodes. Informally, this means that every task has some other task that comes before it in the curriculum. The graph nodes represent subsets of experience (transitions) associated with the intermediate learning tasks. In this general sense, a curriculum defines which intermediate learning tasks should be learned before others to maximise knowledge transfer between tasks. The primary purpose of the curriculum is to help the learner perform better in the final task in the curriculum. Compared to the Supervised Learning formulation of CL in [36], the general idea of ordering experience remains, but the curricula are not limited to a simple sequence of learning tasks. Reference [40] also outlines three key areas of curriculum learning: task generation (how to generate useful intermediate tasks?), sequencing (in what order should we learn the intermediate tasks?), and transfer learning (how should we transfer knowledge between two tasks?).

## 3. Methodology and Experimental Setup

**Figure 2:**
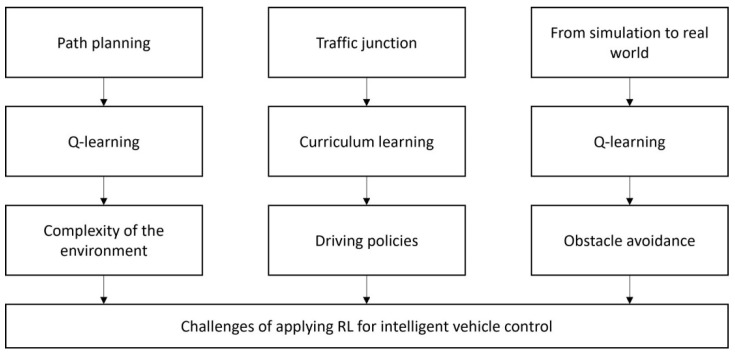
Workflow of the proposed method in this paper.

In this section we describe the environments that are used in the experiments in this work and the platforms used in the proposed sim-to-real approach. The workflow of this project is presented in Figure 2. 

The environment used in the first set of experiments is represented by set of 2D discrete grid world environments $W\subseteq {\mathbb{R}}^{2}$ with size H×W. There are four possible states for each cell in the environment: free cell, occupied by agent, occupied by a static abstract, or occupied by dynamic obstacles, where a set of static and dynamic obstacles is represented as $C_{s}=\left\{s_{1},\ldots ,s_{Ns}\right\}$ and $C_{d}\left(t\right)=\left\{d_{1}\left(t\right),\ldots ,d_{Nd}\left(t\right)\right\}$, respectively. The free cells are denoted by 𝕗 $=\left\{f_{1},\ldots ,f_{Ns}\right\}$. 

The reward function of the model is designed to encourage good moves and discourage wrong actions by the agent. In this task, the agent receives a negative reward when it hits static and dynamic obstacles and then returns to a random starting point. The agent receives a positive reward when it reaches the target point and the episode ends. In this environment, each new episode contains a new unique learning set of static and dynamic obstacles, a new starting point, and a goal point. 

Given the (x,y) position of an agent as result of an action a, the reward function R is defined as:

(3) $$R=\{\begin{array}{c} \begin{array}{c} -5\\ -5 \end{array}\\ 10\\ -1 \end{array}\begin{array}{c} collision\;to\;C_{d}\\ collision\;to\;C_{s}\\ \begin{array}{c} target\;reached\\ penalty\;for\;each\;additional\;move \end{array} \end{array}$$

To compare the transferability of the model to unknown and complex environments, we train the model in different environment settings. The multiple setups are summarized in Table 1. In Section 4 we also present the illustration of the configurations of the experimented environments. In each environment, the number of obstacles is designed in three complexity configurations: easy, moderate, and hard. 

**Figure 3:**
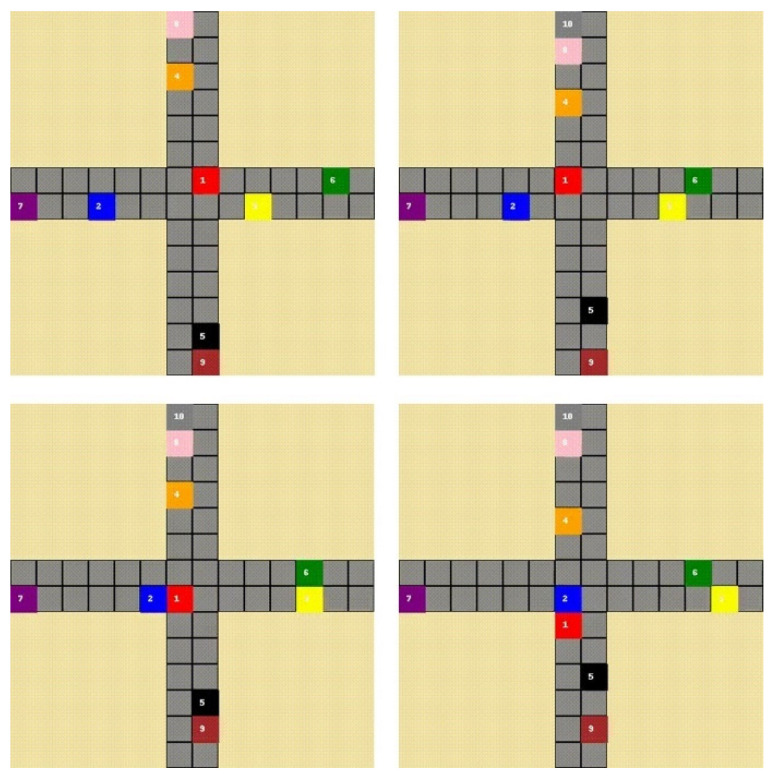
Visualisation of the traffic junction environment with 10 cars. From left to right, top to bottom: observe the cars 1 (red) and 2 (blue) as they approach the intersection and pass it without colliding.

In the second set of experiments in this paper involving CL, we use the Traffic Junction environment as our learning environment, found in a collection of multi-agent learning environments [41]. In this 2D grid world environment, agents are spawned at different ends of a cross-shaped junction. Each agent is assigned a target they must reach by driving forward towards the four-way intersection, making a turn, and driving forward to reach their destination. The agents can take two actions, move forward, or stop, as they move along the pre-defined route towards their destination. However, the reward function is designed to discourage both collisions with other agents and avoid traffic jams, both of which yield a small punishment to the agents. The size of the grid world is 14 by 14 and the number of cars in the environment is 10. The simpler version of the task used for CL is also 14 by 14, but with only 4 cars. For a visual representation of the environment please see Figure 3.

Finally, the experimental setting used in the proposed sim-to-real approach is composed by both a physical platform and a computer simulation to enable transfer of learning between the physical and simulation domains. With the proposed method we try to address the ‘reality gap’ [42].

**Figure 4:**
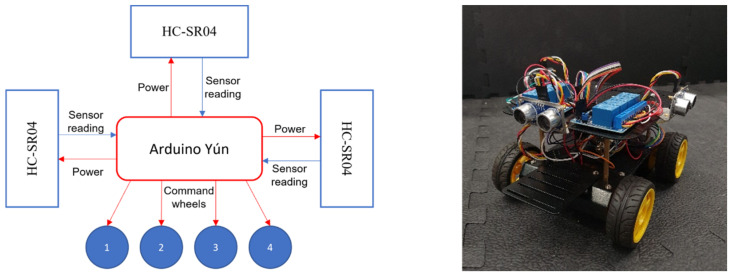
Arduino-controlled Pirate 4WD Mobile Platform (**right**) and diagram illustrating how the main parts of the platform interact with each other (**left**).

For the physical platform, we assembled multiple robots controlled by the microcontroller Arduino Yún. The model of the robots used is the Pirate 4WD Mobile Platform and they use three HC-SR04 ultrasonic sound sensors to gather information from the environment, placed on the front and both sides of the robots (see Figure 4). These sensors will measure the distances to other obstacles in front of them. We have intentionally limited the robots to use just the sound sensors as their only source of information gathering in order to keep them rudimentary and investigate how learning experience can be transferred from simulation to reality in such limited robots. 

It has been discussed before how limited microcontrollers are and how difficult it can be to implement deep neural networks on them due mostly to memory and latency constraints [43,44]. In this sense, we decided to use Q-tables in the presented approach due to their simplicity and ease of transfer and use in the physical platforms controlled by the Arduino Yún. In the simulation implemented we use the Q-learning algorithm [29] to train a Q-table that will be transferred to the physical machines. 

The task implemented in the simulated environment is to roam around and avoid obstacles. The environment is composed by two agents that are trained independently and receive a team reward. In the implementation of the simulation, we reproduce subsequent observations to each agent that would correspond to values gathered by the sound sensors in the physical machines. However, it would be impossible to account in simulation all the infinite possible values that the sensors could measure due to the randomness present in the real world or uncertainties related to the sensors [45,46]. Thus, the key to our method is to create a generalisation of the possible observations received by the ultrasonic sensors and map them to a finite number of values. Hence, in the simulated environment we consider the existence of three possible situations: (1) the front of the agent is free and so it can move forward; (2) the front of the agent is blocked and the distance on the right is greater than on the left; (3) the front of the agent is blocked and the distance on the left is greater than on the right. In the simulation, the agents are trained using RL to react to these scenarios and perform the right actions, resulting in two trained Q-tables and one of them will be exported to the microcontroller. Once the trained Q-table is in the robots, they can roam around and, when they receive an observation, that will be mapped to the generalized values defined before and get the right action from the trained Q-table, avoiding obstacles. Algorithm 1 describes the methodology used for transferring the learnt policies.

**Algorithm 1.** Algorithm used by the Arduino robots to use the transferred policies1.**Set** static map $m\leftarrow \left[f_{s},\;r_{s},\;l_{s}\right]$2.**Input** array of measured distances front, right and left, [f, r, l]
3.**For** each distance *d* in [f, r, l] do4.    map d to value in m, $d\sim \left[f_{s},\;r_{s},\;l_{s}\right]$5.    add *d* to new array of mapped values,
$\left[f_{m},\;r_{m},\;l_{m}\right].insert\left(d\right)$
6.
**End For**
7.**Get** state *s* corresponding to $\left[f_{m},\;r_{m},\;l_{m}\right]$8.**Get**Q-value for state s, $Q_{s}$9.**Output** action $\underset{a}{\mathrm{argmax}}Q_{s}$


## 4. Results and Discussions

In this section we present the results of the experiments performed in this work. The experiments in this paper are three-fold: first, we use Q-learning in a set of different environments to analyse the impact of the environmental complexity in the learning process of the RL agent in path planning tasks for intelligent vehicle control. Second, we show how CL can be applied to MARL driving decision-making scenarios. Third, we investigate a method for transfer RL policies from simulation to reality supported with empirical evidence.

### 4.1. Impact of Environmental Complexity in the Learning Process

**Figure 5:**
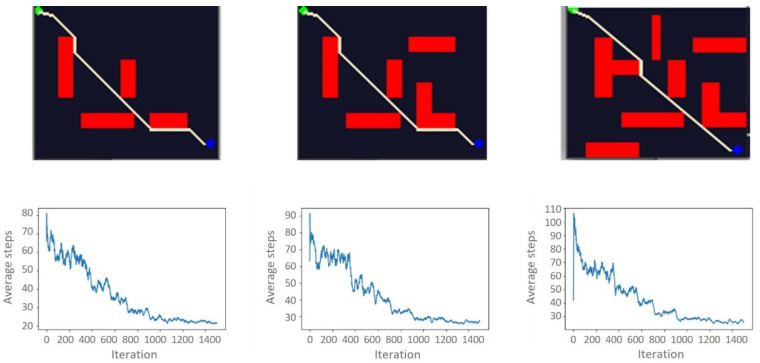
100 × 100 grid-world environments and corresponding number of steps per learning iteration for each configuration. From left to right, easy, moderate, and hard.

**Figure 6:**
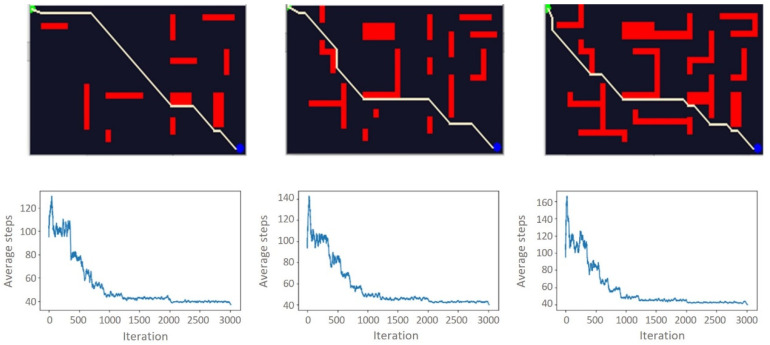
200 × 120 grid-world environments and corresponding number of steps per learning iteration for each configuration. From left to the right, easy, moderate, and hard.

This experimental setup is designed to examine the impact of the environmental complexity on path planning algorithms using RL or, more specifically in this case, Q-learning. Hence, we experimented with six different environments with different complexity configurations, with a 100 × 100 and 200 × 120 grid world systems. Figure 5 and Figure 6 illustrate the 100 × 100 and 200 × 120 grid world systems, respectively. The model was firstly trained in an accessible environment and then is followed by a moderate and a highly complex setup. As illustrated in Figure 5 and Figure 6, based on the complexity of the environment, the model took a different number of iterations to converge. However, we have not found a significant time difference between easy, moderate, and hard configurations for the same environment. Hence, the results suggest that changing the experimental environment to a more complex one will increase the training time more than just adding obstacles to a fixed environment. As the number of obstacles increases, the learning time will also increase.

The results show that the training time of the model is directly proportional to how big the training environment is. On average, it took 19.12 s to train the 100 × 100 and 26.48 s to train the 200 × 120 environments. Table 2 illustrates the total amount of time taken to train the model for the different configurations in each environment.

Environmental contexts in the real world are complex and dynamic. Representing this complexity in simulation is a challenge; however, the real change is the computational cost of training the model. This computational cost is visible in our results. When the environmental complexity increases (Figure 5 and Figure 6), the computational cost also increases. Our paper only considers up to 200 × 120 grid world environments. However, to achieve a more reliable path planning result, the environment needs to be represented in a significantly larger grid world than the one used in this paper.

**Figure 7:**
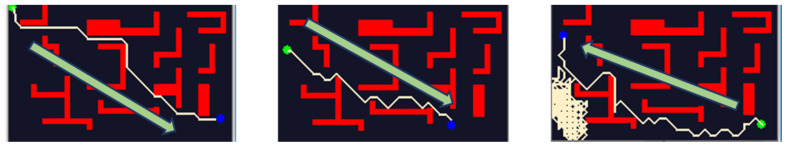
Illustration of the learnt paths for one of the path planning tasks experimented. On the left the training path direction, and on the centre and right the testing learnt path for two different starting and goal places.

Figure 7 illustrates the adaptability problem of the Q-learning based path planning model. One of the significant drawbacks of this method is the lack of generalisation; the agents learn the detailed path of the training route. Our test results show that the model tends to always travel in the direction of the training route without considering the target position. For instance, we trained the model from the top left corner start point to the bottom right corner of the destination.

Our test results show that the model performs better when the destination and start points are similar to the training environment. When we test the model by inverting the start and end points, the model gives lower performance results, suggesting that researchers in RL-based path planning activities need to find a way to train models in multiple routes within a single environment in a short time. As Figure 7 shows, the agent has more advanced knowledge of the environment in the direction of the training path than in another different path.

### 4.2. Curriculum Learning for MARL Driving Decision-Making Scenarios

In this set of experiments, we use a no curriculum baseline and two kinds of curricula in order to negotiate driving decision making policies in a traffic junction task in a MARL setup. For the baseline, we train the policy from a random initialisation for 2 million timesteps. The first kind is the forward curriculum, which is a sequence of tasks (in our case, the simple and target task). First, we train in the simple task, evaluating our performance on the target task periodically. If we see no improvement in the target task for 20 evaluations, we stop training in the simple task and switch to training in the target task. Then, we train in the target task for 2 million time steps. The second kind of curriculum is the sampling curriculum. In a sampling curriculum we sample learning tasks from a distribution of tasks. In our case, we sample the simple task with probability 0.2 and the hard task with probability 0.8. The sampling curriculum may perform better in some environments, for example, in [47] the authors found that a forward curriculum didn’t accelerate learning in one of their tasks, but a sampling curriculum did. We train in the target task for 2 million time steps in total, so approximately 1.6 million time steps in the target and 0.4 million time steps in the simple task.

We use QMIX [48] as the MARL algorithm. QMIX is capable of training policies in a centralised manner that supports decentralised execution. It works by using a mixing network whose weights are derived from the available state information to estimate the joint action values of a team of cooperative agents. More specifically, each agent produces a set of action values based on their local observations, which the mixing network combines to produce the team’s joint action value. The mixing network is designed to make sure the policies behave similarly during both centralised training and decentralised execution phases.

**Figure 8:**
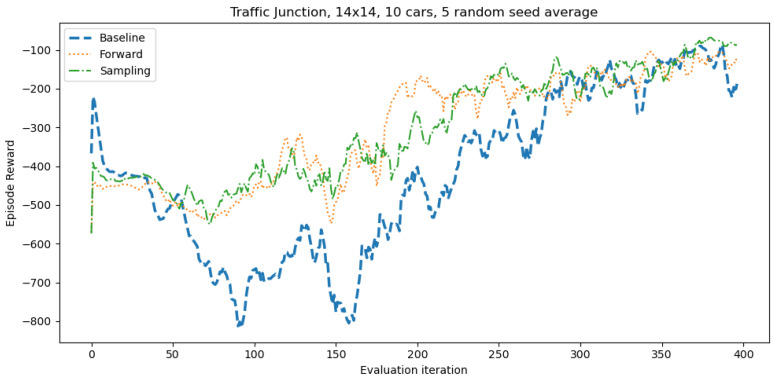
Traffic junction performance using no curriculum, a forward curriculum, and a sampling curriculum.

The results in Figure 8 show that after an initial jump in performance, all policies go through a steady decline in performance. This jump and the following decline are a lot more pronounced when training a policy from scratch, with the highest level of performance around −220, with the policy degrading in performance to around −800. After a temporary increase in performance at around the 90th evaluation iteration the policy degrades again, starting a consistent improvement in performance after the 160th evaluation iteration. From now on, the policy keeps consistently improving with slight but short-lived dips every now and then. The forward curriculum starts out at around −440, declining to −530, after which performance temporarily increases, dips again and sharply increases, similar to the no curriculum baseline. Following this jumpy start, we see a slow and jittery increase in performance up until the last iteration. The sampling curriculum follows a similar path to the forward curriculum, but the fluctuations in performance are less pronounced. Overall, the sampling curriculum shows to be the least unstable with regard to the policy’s performance. Additionally, the sampling curriculum achieves the best performance level during the last iterations of training, followed by the forward curriculum and no curriculum baseline. These results suggests that the sampling curriculum is more efficient compared to others because it trained in the target task for approximately 80% of the time yet achieving better results. This may be due to training in the simple task often enough causes the DNN parameters to be pulled in two directions in parameter space. 80% of the time Gradient Descent (GD) pulls the DNN parameters towards solving the target task, but 20% of the time GD pulls the parameters towards solving the simple task. Assuming there is knowledge in knowing how to solve the simple task that can be used to help learn how to solve the target task, forcing the policy to learn to solve both problems can provide a regularising effect, constraining the possible DNN parameters to a more specific region of parameter space, aiding optimisation. When applying CL there are some factors that need to be accounted in some cases in order to maintain the desired performances. For instance, when using CL, the difficulty of the tasks and subtasks needs to be considered, which might not always be available. Furthermore, there is still some uncertainty when it comes to creating subtasks on-demand and, in some cases, it might be beneficial to filter the type of knowledge being transferred from task to task [40]. However, in scenarios such as the one experimented CL proves to be successful and capable of taking into account these factors.

### 4.3. Sim-to-Real: Transfer of Reinforcement Learning Policies from Simulation to Reality

#### 4.3.1. Simulation Domain

In this section, we show the results of the implementations of Q-learning [29] and DQN (Mnih, et al., 2015) in the simulated environment described in Section 3 that was used to transfer simulation experience to the physical machines. Although deep neural networks can perform better than simple Q-tables in RL when it comes to map states to the corresponding actions, it has been discussed previously how sometimes it can be difficult to implement neural networks in resource limited microcontrollers [43,44]. Therefore, to support the use of Q-tables in this work we perform a set of experiments in simulation. At the same time, the experimental results show how it is possible to minimize the number of collisions with obstacles in a certain environment by using RL.

**Figure 9:**
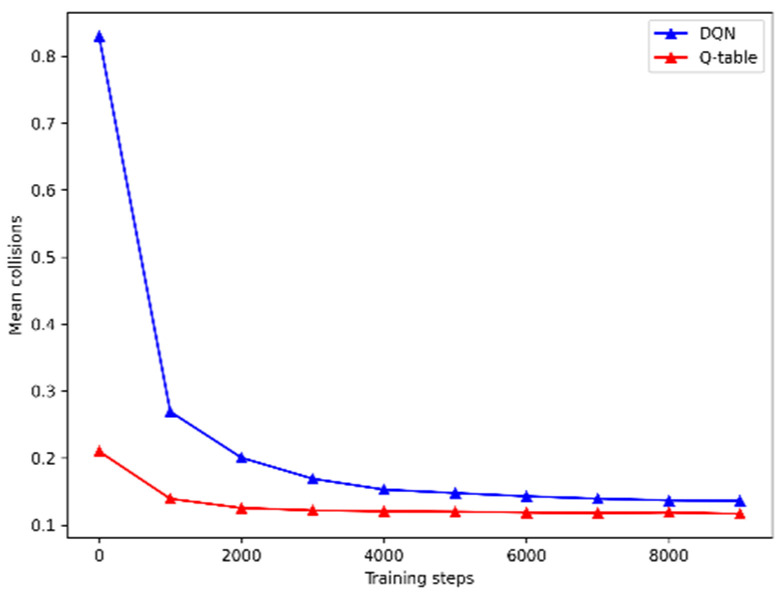
Collisions for the simulation environment described for DQN and Q-table approaches.

In Figure 9 it is illustrated the evolution of the average number of collisions incurred by two agents (the collisions of both agents are added together) per training step over time for simple Q-learning and DQN. As described previously in Section 3, in this task two agents are given successive observations with distances to walls around them. Every time the agents collide with one of the walls each one receives a punishment. In Figure 9 it is possible to see that, although at first both DQN and Q-learning incur in some collisions, after some training time both can minimize the number of collisions, making the performances of the two methods close to each other in further training episodes. Thus, we can conclude that transferring a Q-table instead of a DQN to the physical robots was a reasonable option, since in this setup it would not have impact. Furthermore, in this particular setup, the Q-learning method can minimize the collisions as well as the DQN and in a smaller amount of time.

#### 4.3.2. Real World Domain

To evaluate the fidelity of the proposed transferability approach from simulation to reality, we have implemented a hardcoded policy in a second Arduino Yún controlled robot to roam around and avoid obstacles. Although transferring complex situations learned in simulation to reality is still a big challenge due to multiple constraints such as the randomness in real world events [45,49], the results demonstrate the success of the approach attempted in the presented scenario. By transferring the learned Q-table from the simulation described to reality it was possible to observe a very similar behaviour between both the hardcoded and the robot following the transferred Q-table. They both were able to roam around and avoid obstacles and consequently, they were able to roam in the same area avoiding colliding to each other.

**Figure 10:**
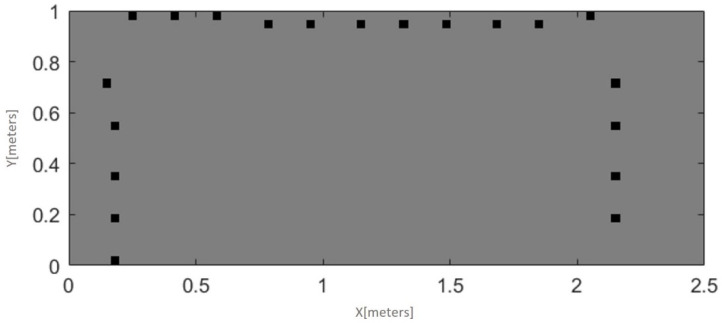
Occupancy grid map built with data collected by one of the Arduino robots corresponding to a rectangular box.

Figure 10 illustrates an occupancy grid map made using data gathered by the robot with the transferred Q-table. The robot was placed inside a large box where it gathered data using the ultrasonic sound sensors. This was possible without any collisions with the walls of the box, following the policy transferred from simulation and, as the figure shows, the box was mapped with a good level of accuracy. 

## 5. Conclusions and Future Work

In this work we have investigated some of the challenges of applying RL within the context of intelligent vehicle control as a real-world application. By referring to common intelligent vehicle control tasks such as path planning using RL, several problems may arise such as the existence of many possible states that the agent should experience, the existence of multiple agents, difficulty of representing states or how should we formulate these safety-critical tasks to be solved by trial-and-error. Considering a use case on path planning, we illustrate how the environmental complexity influences the learning time and the performance of the agents. To mitigate the challenges incurred, we discussed the use of two approaches: a method for transferring RL policies from simulation to reality and a CL based approach to improve learning in driving decision making scenarios. Both these approaches were demonstrated and discussed with reference to use cases pertaining to intelligent vehicle control. Our sim-to-real approach shows to be successful to transfer a collision avoidance policy from a simulated environment to the real world. In a real-world context, our results enhance the importance of simulation to reality experiments by showing that the number of collisions during training time can be minimised in simulation. In our second approach, we demonstrated how CL can be useful when applied to intelligent vehicle control situations. The results showed that using agents with a curriculum to structure the learning process can be beneficial in driving decision making tasks such as a traffic junction task where multiple agents need to negotiate their passages.

In the future, we aim to extend sim-to-real concepts to more complex scenarios, such as dynamic environments and multi decision-making tasks. Furthermore, we intend to investigate how CL can be used together with sim-to-real methods so that the policies trained with CL can be used in reality.

## Figures and Tables

**Table 1:** Configuration of the experimented environments.

Environment	Grid World	Obstacles	Obstacles	Obstacles
Environment 1	100 × 100	easy	moderate	hard
Environment 2	200 × 120	easy	moderate	hard

**Table 2:** Time taken to train the model for the different configurations in each environment.

Environment	Easy	Moderate	Hard
100 × 100	18.23 s	18.41 s	19.52 s
200 × 120	25.11 s	25.41 s	27.44 s

## Data Availability

Not applicable.
